# Incremental Prognostic Value of NT-proBNP Beyond Treadmill Testing for Perioperative Cardiovascular Events in Noncardiac Surgery Candidates: Results from a Multicenter Prospective Cohort

**DOI:** 10.3390/diagnostics16060869

**Published:** 2026-03-14

**Authors:** Jae Seok Bae, Jeong Rang Park, Jae Myoung Lee, Yun-Ho Cho, Jeong Yoon Jang, Yujin Shin, Han Ra Choi, Yong-Lee Kim, Ga-In Yu, Choong Hwan Kwak, Min Gyu Kang, Kye-Hwan Kim, Jin-Yong Hwang, Sung-Eun Park, Young-Hoon Jeong, Jong-Hwa Ahn

**Affiliations:** 1 Division of Cardiology, Department of Internal Medicine, Gyeongsang National University School of Medicine and Gyeongsang National University Changwon Hospital, Changwon 51472, Republic of Korea; baefach@naver.com (J.S.B.);; 2 Department of Internal Medicine, Gyeongsang National University School of Medicine and Gyeongsang National University Hospital, Jinju 52727, Republic of Korea; 3 Department of Radiology, Gyeongsang National University School of Medicine and Gyeongsang National University Changwon Hospital, Changwon 51472, Republic of Korea; 4 CAU Thrombosis and Biomarker Center, Chung-Ang University Gwangmyeong Hospital, Gwangmyeong 14353, Republic of Korea; 5 Department of Internal Medicine, Chung-Ang University College of Medicine, Seoul 06973, Republic of Korea

**Keywords:** non-cardiac surgery, NT-proBNP, treadmill test, risk stratification, major adverse cardiac events, perioperative evaluation

## Abstract

**Background**: Accurate perioperative cardiovascular risk stratification remains challenging in patients undergoing noncardiac surgery. Although treadmill testing (TMT) is widely used for functional assessment, its ability to identify truly high-risk patients is limited. Natriuretic peptides reflect integrated myocardial stress and may provide complementary prognostic information, particularly in patients with abnormal functional test results. **Methods**: In this prospective multicenter observational study, 178 patients with at least one Revised Cardiac Risk Index risk factor undergoing noncardiac surgery were included. All patients underwent preoperative TMT and had available N-terminal pro–B-type natriuretic peptide (NT-proBNP) measurements. The primary endpoint was 30-day major adverse cardiac events (MACE), defined as a composite of cardiac death, nonfatal myocardial infarction, myocardial injury after noncardiac surgery, pulmonary edema with heart failure, and clinically significant arrhythmias. Incremental prognostic value was assessed using the area under the receiver operating characteristic curve (AUC), net reclassification improvement (NRI), and integrated discrimination improvement (IDI), with internal validation using bootstrap resampling. **Results**: At 30 days, 26 patients (14.6%) experienced MACE, of whom seven experienced more than one event. Log-transformed NT-proBNP was independently associated with perioperative events in parsimonious multivariable models. Elevated NT-proBNP, particularly NT-proBNP ≥ 1000 pg/mL, was independently associated with perioperative events after multivariable adjustment. Importantly, the incremental prognostic value of NT-proBNP was most pronounced in patients with a positive TMT, in whom NT-proBNP improved risk discrimination (ΔAUC = +0.09) and reclassification (NRI = 1.00). In contrast, among patients with a negative TMT, the additional prognostic contribution of NT-proBNP was modest and not statistically significant. Subgroup findings should be interpreted cautiously, given the limited number of events. **Conclusions**: Preoperative NT-proBNP provides modest but independent incremental prognostic value beyond treadmill testing, with the greatest impact observed in patients with positive TMT results. Although improvements in discrimination were moderate, NT-proBNP may help refine perioperative risk assessment in selected intermediate- to high-risk patients. These findings support a complementary biomarker-based approach to MACE.

## 1. Introduction

Patients undergoing noncardiac surgery face a considerable risk of perioperative cardiovascular complications, including myocardial infarction, acute heart failure, and cardiovascular death [1,2]. Given the increasing complexity and aging of surgical populations, precise preoperative cardiovascular risk assessment has become a critical component of perioperative care, enabling appropriate perioperative planning, optimization of medical therapy, and informed shared decision-making [3].

Although several clinical risk models have been proposed, the Revised Cardiac Risk Index (RCRI) remains the most commonly applied tool in routine practice because of its simplicity [4]. However, its predictive accuracy is limited in contemporary cohorts, particularly across diverse surgical and patient populations [4,5]. Functional testing, most notably exercise treadmill testing (TMT), has traditionally been incorporated into preoperative evaluation to assess exercise capacity, inducible ischemia, and symptom burden in patients with uncertain functional status [6,7]. Nonetheless, the clinical utility of TMT is constrained by several factors, including baseline electrocardiographic abnormalities, inadequate exercise performance, equivocal test results, and its inability to capture underlying myocardial stress or global cardiovascular vulnerability [8,9,10].

In contrast to functional testing, circulating natriuretic peptides reflect myocardial wall stress and subclinical cardiac dysfunction, providing a global measure of cardiovascular risk. A growing body of evidence has consistently demonstrated that elevated preoperative B-type natriuretic peptide (BNP) or N-terminal pro–B-type natriuretic peptide (NT-proBNP) levels are strongly associated with adverse perioperative cardiovascular outcomes, independent of traditional clinical risk scores and functional test results [11,12,13]. On the basis of this evidence, the Canadian Cardiovascular Society 2022 Focused Update advocates routine preoperative measurement of BNP or NT-proBNP in patients at increased perioperative risk and recommends against the routine use of functional stress testing in the absence of biomarker elevation [14]. This guideline represents a shift toward a biomarker-driven strategy, positioning natriuretic peptides as an initial risk-stratification tool.

Despite these recommendations, TMT continues to be widely used in daily clinical practice because of its accessibility, low cost, and familiarity among clinicians, and it remains embedded in several perioperative evaluation pathways [6,7]. As a result, many patients undergo TMT as part of their preoperative assessment before natriuretic peptide levels are measured. In such settings, it remains uncertain whether NT-proBNP provides incremental prognostic information beyond TMT and whether NT-proBNP can further discriminate perioperative risk according to TMT results.

Accordingly, the present study sought to investigate the incremental prognostic value of NT-proBNP beyond treadmill testing for predicting perioperative cardiovascular events in patients undergoing noncardiac surgery. Furthermore, we aimed to determine whether NT-proBNP refines risk stratification across different TMT results, thereby identifying high-risk perioperative phenotypes characterized by combined ischemic and hemodynamic stress.

## 2. Methods

### 2.1. Study Population

This prospective multicenter observational study was conducted at Gyeongsang National University Changwon Hospital (CGNUH) and Gyeongsang National University Hospital in Jinju (GNUH) between January 2018 and April 2025. A total of 178 consecutive adult patients scheduled for non-cardiac surgery were prospectively enrolled.

Patients were eligible for inclusion if they had at least one clinical risk factor according to the RCRI, including a history of ischemic heart disease, heart failure, or cerebrovascular disease; diabetes mellitus requiring insulin therapy; chronic kidney disease (defined as serum creatinine > 2.0 mg/dL); or if they were scheduled for high-risk surgery, such as intrathoracic, intraperitoneal, or major vascular procedures.

Patients were excluded if they were scheduled for low-risk minor surgery, such as endoscopic, superficial, or ambulatory procedures; required emergency surgery; had contraindications to TMT; or presented with active cardiac conditions, including recent myocardial infarction, decompensated heart failure, severe valvular heart disease, or clinically significant arrhythmias.

All included patients underwent preoperative treadmill testing as part of routine cardiovascular evaluation, and NT-proBNP levels were available for all patients included in the present analysis.

**Figure 1:**
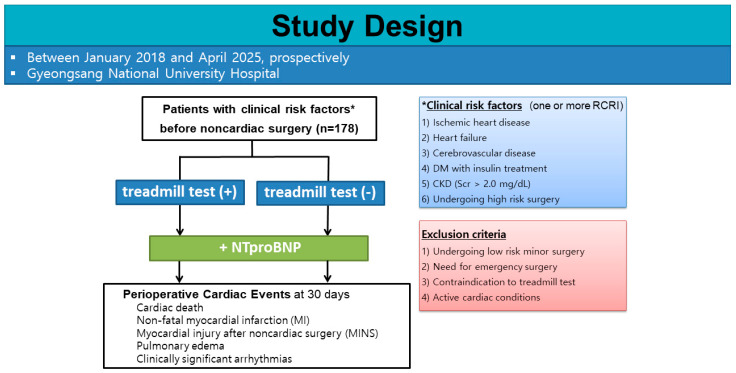
Study Flow Diagram. Flowchart of patient selection and enrollment in the study. A total of 178 patients with at least one Revised Cardiac Risk Index (RCRI) risk factor who underwent preoperative TMT and had available NT-proBNP measurements were included in the final analysis. The primary endpoint was the occurrence of 30-day major adverse cardiac events (MACE), defined as a composite of cardiac death, nonfatal myocardial infarction, myocardial injury after noncardiac surgery, pulmonary edema with heart failure, and clinically significant arrhythmias. *Clinical risk factors were defined according to the Revised Cardiac Risk Index (RCRI), including ischemic heart disease, heart failure, cerebrovascular disease, diabetes mellitus requiring insulin therapy, chronic kidney disease (serum creatinine > 2.0 mg/dL), or high-risk surgery.

The study was approved by the institutional review board of both institutions, and all participants provided written informed consent. The study was registered at ClinicalTrials.gov (Identifier: NCT02250963). The study design and patient selection process are illustrated in Figure 1.

### 2.2. Treadmill Testing

All patients underwent symptom-limited TMT using a standard Bruce protocol [15]. Exercise was terminated upon achievement of the target heart rate (≥85% of the age-predicted maximum), development of limiting chest pain, ≥2 mm horizontal or down-sloping ST-segment depression, or the occurrence of significant arrhythmia or hypotension. A test was considered positive when typical angina developed and/or ≥1 mm ST-segment depression persisted for ≥80 ms after the J point in two contiguous leads [16]. In addition to the binary classification of a positive or negative TMT result, exercise-induced chest pain and dyspnea were prospectively recorded as indicators of functional ischemia. Functional capacity was quantified by the estimated metabolic equivalents (METs) achieved during exercise testing, derived from the maximal workload according to the Bruce protocol. Both continuous MET values and guideline-defined poor functional capacity, defined as METs < 7, were included in the analysis to evaluate the prognostic contribution of functional capacity [17].

### 2.3. NT-proBNP Measurement

Preoperative NT-proBNP levels were measured from venous blood samples obtained within 30 days before the scheduled noncardiac surgery and prior to the surgical procedure. NT-proBNP concentrations were analyzed using a commercially available electrochemiluminescence immunoassay in accordance with the manufacturer’s instructions [18]. NT-proBNP values were analyzed both as a continuous variable (after logarithmic transformation because of right-skewed distribution) and as a categorical variable using prespecified cutoffs based on prior perioperative risk stratification studies and contemporary guideline recommendations [11,14,19]. The ≥1000 pg/mL threshold was prespecified based on prior perioperative literature and guideline recommendations. A Youden-derived cutoff was explored as a secondary analysis and was considered hypothesis-generating [20]. Continuous log-transformed NT-proBNP was used as the primary modeling approach [21].

### 2.4. Outcome Definitions

The primary composite perioperative endpoint consisted of major adverse cardiac events (MACE) occurring within 30 days after noncardiac surgery. MACE was defined as a composite of cardiac death, nonfatal myocardial infarction, myocardial injury after noncardiac surgery, pulmonary edema with heart failure, and clinically significant arrhythmias requiring urgent intervention [12,13]. All patients had scheduled troponin measurements at 6 to 12 h after surgery and on postoperative days 1, 2, and 3, regardless of symptoms, to systematically detect perioperative myocardial injury and minimize detection bias [8,12]. Cardiac death was defined as death attributable to myocardial infarction, malignant arrhythmia, or heart failure. Nonfatal myocardial infarction was diagnosed based on an elevation of cardiac troponin I levels accompanied by ischemic symptoms or new electrocardiographic changes consistent with myocardial ischemia. Myocardial injury after noncardiac surgery (MINS) was defined as a postoperative elevation of cardiac troponin levels in the absence of a non-ischemic cause, according to previously validated criteria [12,19]. Pulmonary edema with heart failure was defined by clinical signs and symptoms requiring medical treatment. Clinically significant arrhythmias were defined as arrhythmic events requiring urgent medical or electrical intervention. All events were identified through structured chart review according to prespecified criteria.

### 2.5. Statistical Analysis

Continuous variables are expressed as mean ± SD or median [IQR] and compared using Student’s *t*-test or Mann–Whitney U test as appropriate. Categorical variables are presented as numbers (%) and compared by chi-square or Fisher’s exact test. Univariable and multivariable logistic regression analyses were used to identify predictors of MACE [22,23]. Each adjusted model included the RCRI in combination with a single predictor of interest, rather than simultaneous inclusion of multiple candidate predictors, in order to preserve model parsimony given the limited number of outcome events [22]. To minimize overfitting given the modest number of outcome events, multivariable models were constructed using a parsimonious strategy, restricting the number of predictors to maintain an acceptable events-per-variable ratio. RCRI was included as a summary clinical risk variable to reduce model complexity. Model calibration was evaluated using calibration slope and intercept, and calibration plots were generated to assess agreement between predicted and observed risk [24]. Collinearity among candidate predictors was assessed using variance inflation factors [25]. Model discrimination was assessed using the area under the receiver operating characteristic (ROC) curve (AUC) and compared using DeLong’s test [26]. Internal validation was performed using bootstrap resampling (1000 iterations) to estimate optimism-corrected discrimination and calibration. Model calibration was evaluated using calibration slope and intercept, and calibration plots were generated to assess agreement between predicted and observed risk. Incremental predictive value was evaluated by net reclassification improvement (NRI) and integrated discrimination improvement (IDI) [27]. Category-free (continuous) NRI was applied in the absence of established perioperative 30-day MACE risk categories; reclassification analyses were considered exploratory. All primary predictors (NT-proBNP, RCRI components, TMT results) were complete for the analyzed cohort, and complete-case analyses were performed without imputation. All statistical analyses were performed using SPSS version 26 (IBM Corp., Armonk, NY, USA) and R version 4.3.2 (R Foundation for Statistical Computing, Vienna, Austria); a two-tailed *p* value < 0.05 was considered statistically significant.

## 3. Results

### 3.1. Baseline Characteristics and Perioperative Cardiovascular Events

A total of 178 patients undergoing noncardiac surgery with available preoperative NT-proBNP measurements were included in the analysis. The mean age was 69.3 ± 8.9 years, and 53.4% were male (Table 1). Cardiovascular risk factors were common: hypertension was present in 73.6%, diabetes mellitus in 53.9%, chronic kidney disease in 18.0%, heart failure in 13.5%, and ischemic heart disease in 37.6% of patients. A prior cerebrovascular accident was present in 29.2%. According to the Revised Cardiac Risk Index (RCRI), 48.9% of patients were classified as class I, 38.2% as class II, 11.2% as class III, and 1.7% as class IV. Most patients underwent intermediate-risk surgery, with orthopedic (38.2%) and intraperitoneal (20.2%) procedures being the most frequent.

### 3.2. Perioperative Cardiovascular Events

Within 30 days after surgery, 26 patients (14.6%) experienced major adverse cardiac events (MACE) (Table 2). The composite endpoint consisted of cardiac death (*n* = 1), nonfatal myocardial infarction (*n* = 12), myocardial injury after noncardiac surgery (*n* = 8), pulmonary edema with heart failure (*n* = 11), and clinically significant arrhythmias (*n* = 2). Among these patients, 7 experienced more than one perioperative cardiovascular event.

### 3.3. Comparison According to Perioperative Events

Baseline clinical risk differed significantly according to event status. Patients with perioperative events had higher RCRI classes compared with those without events (*p* = 0.008) (Table 3). Preoperative NT-proBNP levels were significantly higher among patients who experienced events than among those who did not (median [IQR]: 359.0 [93.5–1568.5] pg/mL vs. 164.1 [48.1–403.5] pg/mL, *p* = 0.038). The proportion of patients with NT-proBNP ≥ 1000 pg/mL was markedly higher in the event group (38.5% vs. 13.2%, *p* = 0.004).

With respect to functional testing, a positive treadmill test was significantly more frequent among patients with events (53.8% vs. 17.1%, *p* < 0.001). Exercise-induced chest pain and dyspnea during treadmill testing were numerically more common in patients with events, although these differences did not reach statistical significance. Mean achieved METs showed a nonsignificant trend toward lower values in the event group (10.92 ± 3.46 vs. 12.13 ± 2.38, *p* = 0.09); however, poor functional capacity (METs < 7) was significantly more prevalent among patients with events (15.4% vs. 2.6%, *p* = 0.022).

### 3.4. Predictors of Perioperative Cardiovascular Events

In unadjusted analyses (Table 4), a positive treadmill test (OR 5.65, 95% CI 2.35–13.62; *p* < 0.001), poor functional capacity (METs < 7) (OR 6.73, 95% CI 1.57–28.87; *p* = 0.010), log-transformed NT-proBNP (OR 1.32, 95% CI 1.04–1.67; *p* = 0.021), and NT-proBNP ≥ 1000 pg/mL (OR 4.13, 95% CI 1.64–10.35; *p* = 0.003) were significantly associated with perioperative events.

After multivariable adjustment, a positive treadmill test (adjusted OR 5.78, 95% CI 2.34–14.26; *p* < 0.001), poor functional capacity (adjusted OR 6.54, 95% CI 1.45–29.46; *p* = 0.014), and NT-proBNP ≥ 1000 pg/mL (adjusted OR 3.40, 95% CI 1.32–8.75; *p* = 0.011) remained independent predictors of perioperative cardiovascular events, whereas log-transformed NT-proBNP showed a borderline association (adjusted OR 1.25, 95% CI 0.98–1.60; *p* = 0.074). Given the modest number of events, confidence intervals were wide for several predictors, reflecting limited precision.

### 3.5. Discriminatory Performance of NT-proBNP

**Figure 2:**
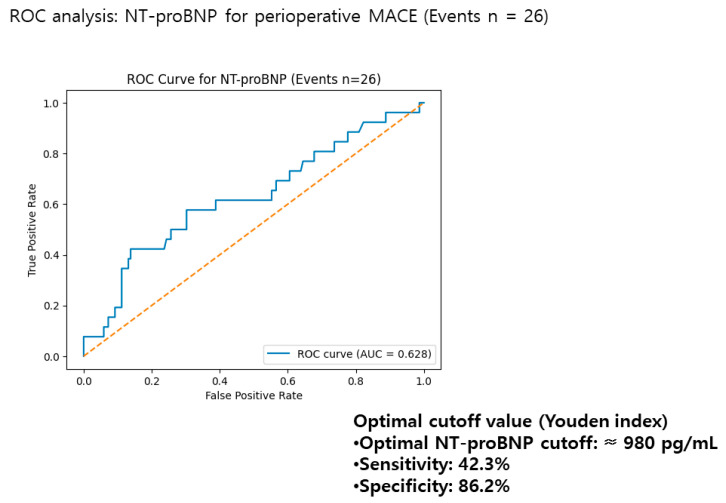
Receiver operating characteristic analysis of NT-proBNP for perioperative cardiovascular events. Receiver operating characteristic (ROC) curve illustrating the discriminatory performance of preoperative NT-proBNP for predicting 30-day major adverse cardiac events (MACE) in patients undergoing noncardiac surgery (events, *n* = 26). The area under the ROC curve (AUC) was 0.628, indicating moderate discriminatory ability. The optimal cutoff value determined by the Youden index was approximately 980 pg/mL, yielding a sensitivity of 42.3% and a specificity of 86.2% for the prediction of perioperative cardiovascular events.

Receiver operating characteristic analysis demonstrated a moderate discriminatory ability of NT-proBNP for predicting perioperative events, with an AUC of 0.628 (Figure 2). The optimal cutoff value derived from the Youden index was approximately 980 pg/mL, yielding a sensitivity of 42.3% and specificity of 86.2%. However, continuous log-transformed NT-proBNP was used as the primary modeling approach, and the Youden-derived cutoff should be considered exploratory.

### 3.6. Incremental Prognostic Value Beyond the RCRI

Model comparison analyses are shown in Table 5. The RCRI-only model demonstrated an AUC of 0.629. Addition of a positive treadmill test significantly improved discrimination (AUC 0.711, *p* = 0.027 vs. RCRI), as well as relative IDI (0.122, *p* < 0.001) and NRI (0.735, *p* < 0.001). In contrast, adding poor functional capacity (METs < 7) resulted in only a modest, non-significant increase in AUC (0.649, *p* = 0.149), despite modest improvements in IDI and NRI.

Addition of NT-proBNP ≥ 1000 pg/mL to the RCRI model produced a modest increase in AUC (0.680, *p* = 0.081) but was associated with significant improvements in reclassification, as reflected by increases in relative IDI (0.047, *p* = 0.009) and NRI (0.506, *p* = 0.005). After bootstrap internal validation, discrimination estimates were modestly attenuated, although overall model performance remained directionally consistent. When both treadmill test positivity and NT-proBNP were added to the RCRI model, discriminatory performance improved further (AUC 0.738, *p* = 0.012 vs. RCRI), with significant gains in IDI and NRI. Reclassification analyses were based on category-free NRI and should be interpreted cautiously in the context of the limited sample size.

### 3.7. Incremental Prognostic Value of NT-proBNP According to Treadmill Test Results

**Figure 3:**
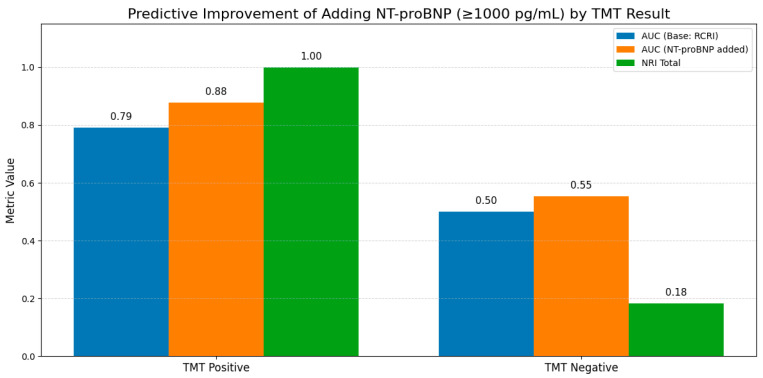
Incremental prognostic value of NT-proBNP according to treadmill test results. Incremental predictive performance of NT-proBNP (≥1000 pg/mL) stratified by treadmill test (TMT) results. Among patients with a positive TMT, the addition of NT-proBNP to the clinical risk model resulted in a substantial improvement in model discrimination and reclassification, with an increase in AUC from 0.79 to 0.88 (ΔAUC = +0.09, *p* = 0.03) and a marked improvement in net reclassification (NRI = 1.00, *p* = 0.01). In contrast, among patients with a negative TMT, the incremental contribution of NT-proBNP was modest and did not reach statistical significance (AUC increase from 0.50 to 0.55, *p* = 0.28; NRI = 0.18, *p* = 0.21). These findings indicate that NT-proBNP provides clinically meaningful incremental prognostic value, particularly in patients with abnormal treadmill test results, refining perioperative risk stratification by identifying a high-risk phenotype characterized by combined ischemic and hemodynamic stress.

Subgroup analyses stratified by treadmill test results are summarized in Table 6 and Figure 3. Among patients with a positive treadmill test, addition of NT-proBNP significantly improved discrimination (AUC increase from 0.79 to 0.88, ΔAUC = +0.09, *p* = 0.03), as well as relative IDI (0.189, *p* = 0.01) and NRI (1.00, *p* = 0.01). In contrast, among patients with a negative treadmill test, NT-proBNP provided only modest and non-significant improvements in discrimination (AUC increase from 0.50 to 0.55, *p* = 0.28) and reclassification (NRI = 0.18, *p* = 0.21). Given the limited number of events within subgroups, these findings should be considered exploratory and hypothesis-generating.

## 4. Discussion

In this multicenter prospective cohort of patients undergoing noncardiac surgery, we demonstrated that preoperative NT-proBNP provides incremental prognostic information beyond TMT for the prediction of MACE. Several key findings emerge from this study. First, elevated NT-proBNP was independently associated with MACE after adjustment for established clinical risk factors and functional testing parameters. Second, NT-proBNP improved risk reclassification beyond the RCRI, even when gains in conventional discrimination metrics were modest. Third, the incremental prognostic value of NT-proBNP appeared to be more pronounced among patients with a positive TMT, suggesting potential clinical relevance in this subgroup; however, this subgroup finding should be considered exploratory and hypothesis-generating given the limited number of events.

### 4.1. NT-proBNP as an Integrated Marker of Perioperative Risk

Functional stress testing has long played a central role in preoperative cardiovascular evaluation by identifying inducible ischemia and assessing functional capacity, as emphasized in contemporary perioperative guidelines [1,2]. However, treadmill testing primarily reflects exercise-induced ischemic burden and may not fully capture underlying myocardial stress, subclinical ventricular dysfunction, or global cardiovascular vulnerability [8,11]. In contrast, natriuretic peptides reflect integrated hemodynamic stress and neurohormonal activation, providing a broader measure of cardiovascular risk that extends beyond ischemia alone [16,21].

Consistent with prior perioperative studies, we observed significantly higher NT-proBNP levels among patients who experienced perioperative cardiovascular events. These findings align with previous meta-analyses demonstrating the prognostic value of BNP and NT-proBNP in the perioperative setting [16,21]. However, given the modest event count and model complexity, these findings should be interpreted with appropriate caution. Internal validation using bootstrap resampling demonstrated some degree of optimism in model performance, although discrimination remained acceptable after optimism correction [23]. Beyond perioperative surgical populations, natriuretic peptides have demonstrated consistent prognostic value across a broad spectrum of acute cardiovascular stress states. A recent study published in the American Journal of Emergency Medicine reported that elevated natriuretic peptide levels were independently associated with adverse neurological and survival outcomes following cardiac arrest [19]. Although conducted in a distinct clinical context, this study reinforces the concept that NT-proBNP reflects global myocardial stress and systemic hemodynamic burden rather than isolated ischemia. Taken together, these findings support the broader biological plausibility of NT-proBNP as an integrative risk marker in acute and perioperative cardiovascular settings.

### 4.2. Incremental Value Beyond Treadmill Testing and Clinical Risk Scores

Our results extend existing evidence by demonstrating that NT-proBNP provides incremental prognostic information beyond treadmill testing and established clinical risk scores. While improvements in AUC were modest and in some comparisons did not reach statistical significance, NT-proBNP contributed to improved reclassification metrics (IDI and NRI) [26]. It is well recognized that AUC is relatively insensitive to incremental changes in risk prediction, particularly when baseline model performance is moderate [26]. Importantly, category-free NRI was used in the present study, and such metrics may overestimate incremental value in small samples with limited event counts [27]. Therefore, the reclassification findings should be considered supportive rather than definitive evidence of clinical superiority. When both treadmill testing and NT-proBNP were incorporated into the clinical risk model, predictive performance improved modestly. Nevertheless, given the relatively low events-per-variable ratio, we adopted a parsimonious modeling strategy consistent with established methodological recommendations [22] and performed bootstrap internal validation to mitigate overfitting and estimate optimism-adjusted performance [23]. Even after these adjustments, NT-proBNP retained incremental prognostic value, although effect sizes were attenuated compared with the apparent (non-validated) estimates. In addition to discrimination and reclassification metrics, model calibration was formally assessed to ensure agreement between predicted and observed risk. Calibration slopes derived from bootstrap internal validation demonstrated acceptable agreement, although some degree of optimism was observed, as expected in models derived from modest event counts [23]. These findings underscore the importance of internal validation and calibration assessment when evaluating incremental biomarkers in small- to intermediate-sized cohorts [23].

### 4.3. Endpoint Considerations

The composite endpoint included cardiac death, nonfatal myocardial infarction, MINS, pulmonary edema, and clinically significant arrhythmias. These components differ in severity and pathophysiology, and endpoint heterogeneity may influence effect estimates. Although this composite approach aligns with prior perioperative studies [12,13,14,18], interpretation of results should account for the varying clinical weight of individual components. The use of systematic postoperative troponin surveillance at prespecified intervals (6–12 h and postoperative days 1–3) further strengthened endpoint ascertainment and reduced the likelihood of detection bias [12,13]. Uniform biomarker monitoring across the cohort enhances confidence that MINS was consistently identified, independent of symptom-driven testing patterns, consistent with contemporary perioperative myocardial injury frameworks [18].

### 4.4. Clinical Implications and Guideline Relevance

Our findings are broadly consistent with Canadian Cardiovascular Society recommendations, which emphasize the role of natriuretic peptides in perioperative risk assessment [12]. Rather than replacing treadmill testing, NT-proBNP may help refine risk estimation, particularly in patients with abnormal functional test results. However, the modest improvements in discrimination metrics underscore that NT-proBNP should be considered a complementary tool within a broader clinical framework, rather than a standalone determinant of management decisions. Although improvements in AUC were modest, incremental biomarkers may influence clinical decision-making in ways not fully captured by discrimination statistics alone [26]. Decision curve analysis has been proposed as a complementary approach for evaluating clinical net benefit beyond conventional discrimination metrics [28]. Future studies incorporating decision curve analysis and external validation would therefore be valuable to clarify clinical utility and quantify the potential net benefit of NT-proBNP-guided perioperative strategies [28,29]. Furthermore, economic and health-system implications should also be considered. Cost-effectiveness modeling may help determine whether biomarker-guided strategies meaningfully alter perioperative management pathways and improve patient-centered outcomes [30].

## 5. Limitations

Several limitations should be acknowledged. First, although prospective and multicenter in design, the sample size was modest and the number of events limited, increasing the risk of overfitting and reducing the precision of effect estimates. Given the relatively low events-per-variable ratio, model coefficients may be unstable despite the use of a parsimonious modeling strategy, and apparent performance estimates may overstate true predictive ability. Second, despite implementing parsimonious modeling and bootstrap internal validation to estimate optimism-corrected performance, internal validation cannot substitute for independent external validation, and replication in larger, contemporary perioperative cohorts is necessary before broad clinical application. Third, NT-proBNP was measured at a single preoperative time point, and serial biomarker assessment was not performed. Dynamic perioperative changes in natriuretic peptide concentrations, which may carry additional prognostic information, were therefore not captured in this analysis. Fourth, the cohort included only patients with at least one RCRI risk factor, limiting generalizability to lower-risk surgical populations. Accordingly, the incremental value of NT-proBNP beyond treadmill testing may differ in patients without established clinical risk factors or in minimally invasive surgical settings. Fifth, although systematic postoperative troponin surveillance was performed, formal blinded endpoint adjudication by an independent committee was not conducted, and residual misclassification cannot be completely excluded. Sixth, the composite endpoint incorporated events of varying severity and pathophysiology. While consistent with prior perioperative studies, such heterogeneity may influence effect estimates and complicate interpretation of the clinical magnitude of benefit. Finally, perioperative management was not standardized according to NT-proBNP levels, and whether biomarker-guided strategies improve outcomes remains to be determined. The present study was observational in nature; therefore, causal inference cannot be established, and prospective interventional trials are required to determine whether NT-proBNP-guided perioperative strategies meaningfully improve patient-centered outcomes.

## 6. Conclusions

In patients undergoing noncardiac surgery, NT-proBNP was independently associated with 30-day major adverse cardiac events and provided incremental prognostic information beyond TMT and clinical risk assessment. Although improvements in discrimination were modest and findings warrant cautious interpretation given the limited sample size and event count, NT-proBNP may contribute to more refined perioperative risk stratification—particularly among patients with positive TMT results. These findings support further investigation of biomarker-integrated approaches to perioperative cardiovascular risk assessment in larger cohorts with external validation.

## Figures and Tables

**Table 1:** Characteristics of the study population.

Variable	Patients (*n* = 178)
Age (year)	69.3 ± 8.9
Body mass index (kg/m^2^)	23.0 ± 3.5
Male gender	95 (53.4%)
Current smoking habits	46 (25.8%)
Diabetes	96 (53.9%)
Hypertension	131 (73.6%)
Chronic kidney disease	32 (18.0%)
Heart failure	24 (13.5%)
Ischemic heart disease	67 (37.6%)
Previous PCI	28 (15.7%)
Cerebrovascular accident	52 (29.2%)
Revised cardiac risk index score	
I	87 (48.9%)
II	68 (38.2%)
III	20 (11.2%)
IV	3 (1.7%)
Type of surgery	
High risk: vascular	27 (15.2%)
Intermediate risk	
- Intra-peritoneal	36 (20.2%)
- Intra-thoracic	9 (5.1%)
- Orthopedic	68 (38.2%)
- Major spine	30 (16.9%)
- Head and neck	8 (4.5%)

Values are mean ± SD or *n* (%). PCI = percutaneous coronary intervention.

**Table 2:** Perioperative Cardiovascular Events and All-Cause Mortality.

Variable	Patients (*n*)
**Perioperative cardiac events**	**26**
- Cardiac death	1
- Non-fatal myocardial infarction	12
- Myocardial injury after noncardiac surgery	8
- Pulmonary edema with heart failure	11
- Clinically significant arrhythmias requiring urgent intervention	2

Data are presented as number of patients. Seven patients experienced more than one perioperative cardiovascular event.

**Table 3:** Baseline results of CTA and TMT by Events vs. No Events.

Variable	Total Patients (*n* = 178)	No Events (*n* = 152)	Events (*n* = 26)	*p*
Revised cardiac risk index score				0.005
I	87 (48.9%)	80 (52.6%)	7 (26.9%)	
II	68 (38.2%)	56 (36.8%)	12 (46.2%)	
III	20 (11.2%)	15 (9.9%)	5 (19.2%)	
IV	3 (1.7%)	1 (0.7%)	2 (7.7%)	
NT-proBNP (pg/mL)				
Median (IQR)	188.3 (52.4–480.0)	164.1 (48.1–403.5)	359.0 (93.5–1568.5)	0.038
NT-proBNP ≥ 1000 pg/mL	30 (16.9%)	20 (13.2%)	10 (38.5%)	0.004
Treadmill Test (TMT)				
Positive TMT result	40 (22.5%)	26 (17.1%)	14 (53.8%)	<0.001
Chest pain during TMT	25 (14.0%)	19 (12.5%)	6 (23.1%)	0.12
Dyspnea during TMT	14 (7.9%)	10 (6.6%)	4 (15.4%)	0.17
Functional capacity				
METs	11.95 ± 2.59	12.13 ± 2.38	10.92 ± 3.46	0.09
METs < 7	8 (4.5%)	4 (2.6%)	4 (15.4%)	0.022

Values are mean ± SD or n (%).

**Table 4:** Unadjusted and Adjusted Odds Ratios for Perioperative Events.

Predictors	Unadjusted OR	95% CI	*p*-Value	Adjusted OR (for RCRI)	95% CI	*p*-Value
Revised cardiac risk index ≥ II	3.02	1.20–7.59	0.019	-	-	-
Positive TMT result	5.65	2.35–13.62	<0.001	5.78	2.34–14.26	<0.001
Chest pain during TMT	2.10	0.75–5.89	0.16	2.40	0.82–6.97	0.11
Dyspnea during TMT	2.58	0.74–8.95	0.14	2.51	0.70–8.98	0.16
METs < 7	6.73	1.57–28.87	0.010	6.54	1.45–29.46	0.014
NT-proBNP (log-transformed)	1.32	1.04–1.67	0.021	1.25	0.98–1.60	0.07
NT-proBNP ≥ 1000 pg/mL	4.13	1.64–10.35	0.003	3.40	1.32–8.75	0.011

Models were constructed using parsimonious selection with adjustment for RCRI only. The events-per-variable ratio was 8.7. Bootstrap internal validation (1000 resamples) was performed to estimate optimism. Adjusted models were constructed by including the RCRI and one additional predictor per model.

**Table 5:** Model Discrimination, Reclassification, and Internal Validation.

Models	Apparent AUC	Optimism-Corrected AUC	Calibration Slope	*p* (vs. RCRI)	Relative IDI	*p* (IDI)	NRI (Total)	*p* (NRI)
RCRI (≥II) only	0.629	0.629	1.197	Reference	Reference	-	Reference	-
RCRI (≥II) + Positive TMT	0.711	0.710	1.032	0.027 *	0.122	<0.001 *	0.735	<0.001 *
RCRI (≥II) + METs < 7	0.649	0.645	1.004	0.150	0.052	0.027 *	0.255	0.031 *
RCRI (≥II) + NT-proBNP ≥ 1000	0.680	0.675	1.015	0.081	0.047	0.009 *	0.506	0.005 *

Apparent AUC values are based on the original sample. Optimism-corrected AUC and calibration slope were estimated using bootstrap internal validation with 1000 resamples. NRI represents category-free net reclassification improvement. Given the limited number of events, reclassification metrics should be interpreted cautiously (* *p* < 0.05).

**Table 6:** Incremental Prognostic Value of NT-proBNP (≥1000 pg/mL) According to TMT Result (Exploratory; Bootstrap 800 resamples).

Scheme	AUC (Base)	AUC (With NT-proBNP)	Optimism-Corrected AUC (with NT-proBNP)	Calibration Slope (With NT-proBNP)	ΔAUC	IDI	NRI (Total)
TMT Positive (*n* = 40, events = 14)	0.790	0.880	0.874	1.161	+0.09 *	0.189 *	1.00 *
TMT Negative (*n* = 138, events = 12)	0.500	0.550	0.461	0.294	+0.05	0.005	0.180

Subgroup analyses are exploratory and were not adjusted for multiple comparisons. Optimism-corrected AUC and calibration slope were estimated using bootstrap internal validation (800 resamples). Reclassification metrics are category-free and may be unstable in small samples. * statistically significant (*p* < 0.05).

## Data Availability

The original contributions presented in this study are included in the article. Further inquiries can be directed to the corresponding author.
